# Xanthogranulomatous Pyelonephritis and Plummer-Vinson Syndrome: A Case Report Exploring Potential Connections in a Single Patient

**DOI:** 10.7759/cureus.69097

**Published:** 2024-09-10

**Authors:** Jagadeswar Kakumani, Amukthamalyada Koduri, Prem Balaji Reddy Lankapothu, Magesh Kumar S

**Affiliations:** 1 Internal Medicine, Saveetha Medical College and Hospitals, Saveetha Institute of Medical and Technical Sciences, Saveetha University, Chennai, IND; 2 General Medicine, Saveetha Medical College and Hospitals, Saveetha Institute of Medical and Technical Sciences, Saveetha University, Chennai, IND

**Keywords:** chronic pyelonephritis, dysphagia', hematuria, plummer-vinson syndrome, xanthogranulomatous pyelonephritis

## Abstract

Xanthogranulomatous pyelonephritis (XGP) and Plummer-Vinson Syndrome (PVS) are two rare disorders that pose considerable diagnostic difficulties mainly because their signs overlap and are multifaceted. XGP is a severe form of pyelonephritis imitating cancer, whereas PVS is defined by dysphagia, iron deficiency anemia, and esophageal webs. This article presents the case of a 53-year-old female with a previous history of renal calculi and multiple transfusions who presented with dysphagia, flank pain, and hematuria. Findings from investigations showed severe anemia, a renal lesion suggesting malignancy, and GI findings pointing to the presence of PVS. The coexistence of XGP and PVS in this patient highlights the need for careful differential diagnosis and the importance of a multidisciplinary approach in managing patients with rare overlapping syndromes. Furthermore, this example shows how chronic infection, malnutrition, and their potential for leading to neoplasms connect. In summary, one should recognize that the possibility these two conditions can coincide is paramount for accurate diagnosis and effective management. Lastly, this case demonstrates that comprehensive assessment can only be achieved through coordinated care addressing both direct effects as well as secondary complications due to such uncommon diseases

## Introduction

Xanthogranulomatous pyelonephritis (XGP) is a distinctive and aggressive form of chronic pyelonephritis that is characterized by a pronounced granulomatous reaction with xanthoma cells, which are lipid-laden macrophages formed as a response to chronic infection [1]. This condition accounts for about 1% of all cases of pyelonephritis, predominantly affecting middle-aged women, though pediatric cases have also been reported, highlighting its potential impact across age groups [2]. The typical etiological agent in XGP is obstructing renal calculi, which leads to stasis and subsequent infection, predominantly with Proteus mirabilis, Escherichia coli, and other urease-producing organisms [3]. Infection becomes chronic through this blockage and the body responds to it using macrophages. Over time, these macrophages accumulate lipids and become xanthoma cells. These inflammatory processes lead to renal parenchymal destruction as the condition progresses and may extend beyond the kidney causing complications like perinephric abscesses, reno-colic fistulas, or even rare occurrences of psoas abscesses [4]. Diagnosis can be complicated by extensive clinical and radiological changes that may resemble neoplasms but are often not necessary to perform nephrectomies [5]. Plummer-Vinson Syndrome (PVS), also known as Paterson-Brown-Kelly Syndrome, primarily affects middle-aged women however it is more uncommon among men or younger populations [6]. Dysphagia iron-deficiency anemia esophageal webs make up a triad characteristic of PVS [7]. The prevalence of PVS has seen a significant decline particularly in developed countries likely attributable to improved nutritional standards such as better access to dietary iron. Although not fully understood, the pathogenesis of PVS is largely related to iron deficiency. Deficiency of this metal results in atrophy of the mucosa and the formation of thin membranes in the upper part of the esophagus. These stairs may cause dysphagia thereby increasing the risk for squamous cell carcinoma in the esophagus as well as the pharynx [8]. Continuous anemia seen in PVS further worsens fatigue and weakness, worsening overall well-being [9]. The interaction between XGP and PVS presents a unique clinical scenario. Iron sequestration by the reticuloendothelial system leading to increased iron deficiency due to chronic inflammation from XGP can subsequently predispose or exacerbate manifestations of PVS [10]. Patients with either condition must be managed comprehensively since coexistence may complicate the clinical picture and therapeutic strategies.

## Case presentation

**Figure 1:**
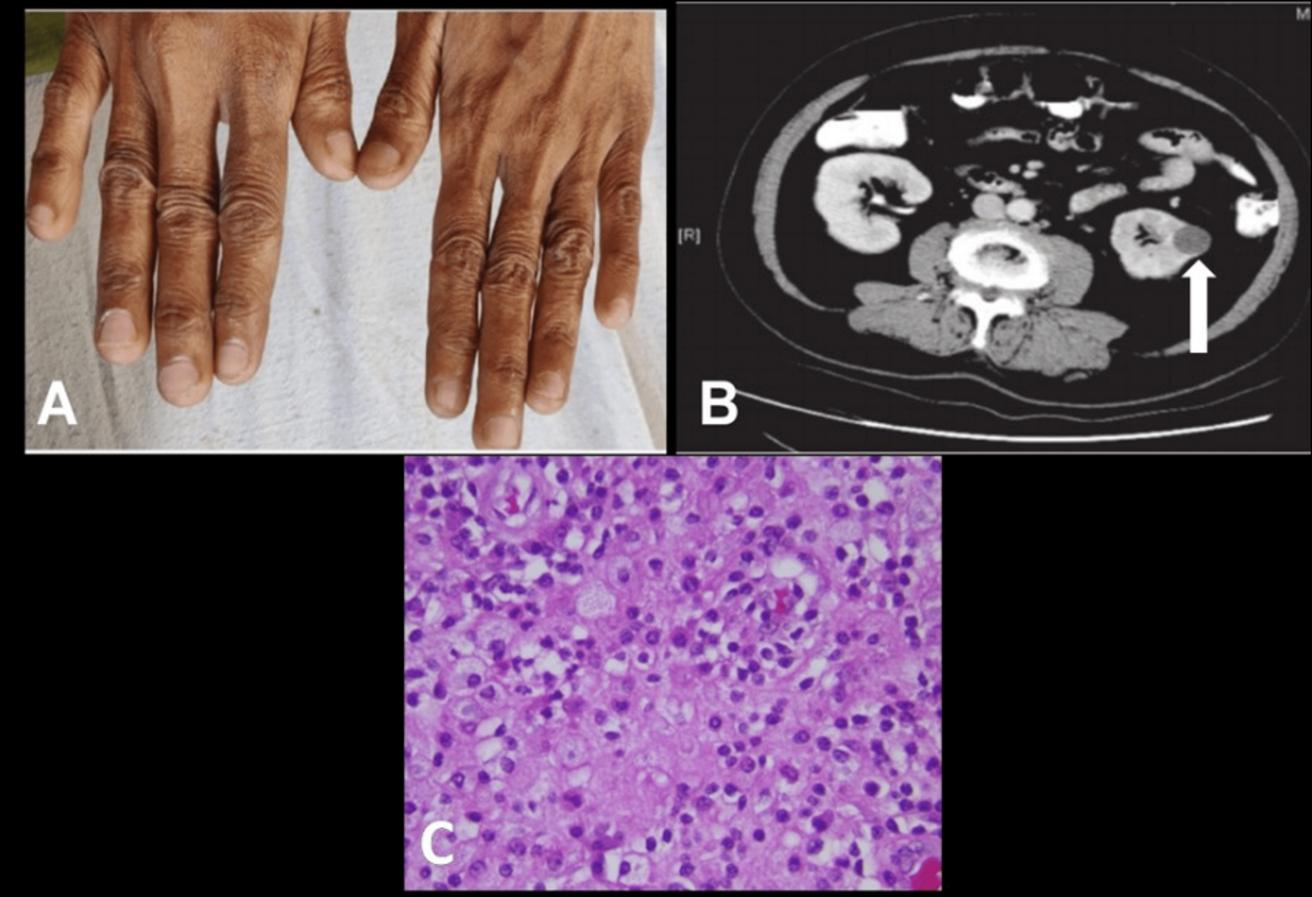
(A) Koilonychia; (B) computed tomography (CT) of the kidneys, ureters, and bladder showing left renal cyst (arrow); (C) histopathology of xanthogranulomatous pyelonephritis shows foamy macrophages, plasma cells, and chronic inflammation.

A 53-year-old woman with no significant comorbidities presented to the outpatient department with worsening progressive dysphagia for solids, which had been developing over the past year but had notably intensified in the last few months. This worsening of dysphagia coincided with the emergence of renal complications, prompting an investigation into a possible link between these conditions. The patient also reported episodes of hematuria, nausea followed by vomiting, and severe flank pain radiating to her back. Her medical history included chronic anemia, which had necessitated multiple blood transfusions, and bilateral renal stones. Two months prior, she had undergone double J stenting. On physical examination, she appeared malnourished with marked pallor, angular cheilitis, a dark red tongue, and spoon-shaped nails, all indicative of iron deficiency (Figure 1A). Abdominal palpation revealed tenderness in the right flank with palpable hardness likely due to previous stent placements, though no masses were detected. Despite the absence of peritoneal irritation signs, the patient experienced significant pain. Initial blood tests revealed severe anemia with a hemoglobin level of 8.2 mg/dL and microcytic hypochromic anemia on peripheral smear. Occult blood in her stool suggested ongoing gastrointestinal bleeding. Urinalysis showed approximately 15-20 abnormal red blood cells per high power field, and urine culture grew E. coli, indicating an active urinary tract infection (Table 1). Upper gastrointestinal endoscopy revealed postcricoid webbing, grade B esophageal candidiasis, a relaxed lower esophageal sphincter, and pangastritis, findings characteristic of PVS. A CT KUB scan identified a heterogeneous lesion measuring 4.6 cm x 4.0 cm, with internal septations and calcifications, classified as Bosniak type IV (Figure 1B). This initially raised concerns for malignancy due to its involvement with the descending colon and the formation of a reno-colic fistula. However, a diagnostic renal biopsy revealed lipid-laden macrophages (Figure 1C), confirming XGP and excluding renal cell carcinoma. The patient was prescribed antibiotics according to culture sensitivity results and advised to undergo nephrectomy. However, she was unwilling to proceed with the surgery and chose to discharge herself against medical advice.

**Table 1:** Depicts the laboratory values. TIBC, total iron-binding capacity

Test	Result	Normal range
Hemoglobin	8.2 mg/dL	13.5-17.5 g/dL
Hematocrit	26%	41%-53%
Red blood cell (RBC) count	3.8 x 10^6^/µL	4.7-6.1 x 10^6^/µL
Mean corpuscular volume (MCV)	65 fL	80-100 fL
Peripheral smear	Microcytic hypochromic RBC	
White blood cell (WBC) count	16 x 10^3^/µL	4.5-11.0 x 10^3^/µL
Platelet count	220 x 10^3^/µL	150-450 x 10^3^/µL
Erythrocyte sediment rate (ESR)	56 mm/hour	0-20 mm/hour
C-reactive protein (CRP)	210 mg/dL	0.3-1.0 mg/dL (mild), 1.0-10.0 mg/dL (moderate), >10.0 mg/dL (marked elevation), >50.0 mg/dL (severe)
Serum procalcitonin (PCT)	3.0 ng/mL	Normal/Low Risk: Less than 0.1 ng/mL; slightly elevated: 0.1-0.5 ng/mL. This may suggest a localized infection but is not usually indicative of severe systemic infection or sepsis. Moderately Elevated: 0.5-2 ng/mL, This can indicate a higher likelihood of systemic infection or sepsis, particularly if other clinical signs are present. Highly elevated: >2 ng/mL. This strongly suggests sepsis or a severe bacterial infection.
Serum iron	<10	65-176 µg/dL (11.6-31.5 µmol/L)
Serum ferritin	20.6	24-336 ng/mL (24-336 µg/L)
Serum TIBC	514	261-462
SERUM vitamin B 12	523	Normal Vitamin B12 levels: 200-900 pg/mL (148-665 pmol/L); potential deficiency: <200 pg/mL (<148 pmol/L); borderline deficiency: 200-300 pg/mL (148–221 pmol/L)
Stool occult blood	Positive	
Serum urea	19	Adults: 6-20 mg/dL (2.1-7.1 mmol/L)
Serum creatinine	0.5	0.74-1.35 mg/dL (65-120 µmol/L)
Serum uric acid	4.2	3.4-7.0 mg/dL (202-416 µmol/L)
Urine Ph	7.9	4.6-8.0
Urine RBC	16	0-2/hpf
Urine WBC	20	0-5/hpf
Urine protein	1+	-
Urine bacteria	Present	
Urine culture	Escherichia coli (>1,000,000 cfu)	

## Discussion

The combination of XGP and PVS highlights the potential connections between these rare conditions. This discussion explores the possible interplay between the pathophysiological mechanisms of each condition, the diagnostic considerations, and the clinical implications of their rare co-occurrence. XGP is a severe chronic form of pyelonephritis, which can be misdiagnosed due to its similar presentation with other more sinister pathologies like renal cell carcinoma [11]. The long-standing obstruction that characterizes XGP is usually caused by renal stones, which creates an environment conducive to chronic infection leading to the granulomatous destruction seen in these tissues [12]. Clinicians may mistake the gross appearance of kidneys affected by this condition as indicative of a malignant neoplasm thus; misleading radiologists too. The presence of xanthoma cells constitutes the hallmark granulomatous response, characterized as lipid-laden macrophages that result from lysed kidney cells [13]. XGP has potentially fatal complications such as the formation of fistulas like our patient who had reno-colic fistulae. This complication occurs when the granulomatous process extends beyond renal parenchyma to involve adjacent organs such colon for this particular case. Such kind of a fistula further complicates matters making it necessary to exhaustively diagnose and manage the situation [14]. On the other hand, PVS is an uncommon disease characterized by dysphagia, iron-deficiency anemia, and esophageal webs often seen among middle-aged women in developing countries where nutrition standards are poorer than developed ones. In upper alimentary tract mucosa chronic iron deficiency leads to atrophic changes which results in the formation of webs capable of obstructing the esophagus hence causing PVS [15].In addition to her nutritional status, her ongoing gastrointestinal bleeding resulting positive stool occult blood test could worsen this chronic iron deficiency. PVS, however, has made her presentation more complex than those of merely symptomatic overlap and increased risk for esophageal carcinoma, which necessitates heightened vigilance in surveillance and management. This is an exceedingly unusual case where XGP and PVS have occurred together in the same patient; hence being managed will be a unique situation. Delays in diagnosis or mismanagement can occur if both diseases are manifested through symptoms that may mask or mimic each other. For instance, since chronic inflammation with XGP leads to increased metabolic demands as well as nutrient sequestration by inflammatory cells it would contribute towards the severity of PVS through exacerbation of iron deficiency [16]. Moreover, treatment approaches to these two conditions can be convoluted as well. Surgical intervention is usually necessary for XGP, especially when there are complications such as the formation of fistulas or massive kidney destruction. However, the management of PVS mainly addresses issues concerning anemia and closely monitors for possible malignant changes within the esophagus associated with it. To deal effectively with both conditions while watching out for their respective complications therefore requires collaborative care between gastroenterologists and nephrologists/urologists. As dysphagia in many patients resolves with just iron supplementation, dysphagia caused by more advanced disease is unlikely to respond to medical management alone and, thus, is managed with endoscopic dilation. XGP is initially treated with antibiotics and percutaneous drainage, and if the patient improves, nephrectomy (partial/total) may be considered; otherwise, surgery is performed if there is no clinical improvement.

## Conclusions

The concurrent presence of XGP and PVS in this patient suggests a potential link between the two conditions. This case underscores the importance of exploring possible interconnections between such rare overlapping conditions in clinical practice.
